# Carbon/Attapulgite Composites as Recycled Palm Oil-Decoloring and Dye Adsorbents

**DOI:** 10.3390/ma11010086

**Published:** 2018-01-06

**Authors:** Guangyan Tian, Wenbo Wang, Yongfeng Zhu, Li Zong, Yuru Kang, Aiqin Wang

**Affiliations:** 1 Key Laboratory of Clay Mineral Applied Research of Gansu Province, Center for Eco-Material and Green Chemistry, Lanzhou Institute of Chemical Physics, Chinese Academy of Sciences, Lanzhou 730000, China; sunshine_015@126.com (G.T.); zhuyf851013@163.com (Y.Z.); zongli@licp.cas.cn (L.Z.); yurukang@licp.cas.cn (Y.K.); 2 Key Laboratory of Special Function Materials for Ecological Environment and Information, Hebei University of Technology, Ministry of Education, Tianjin 300130, China; 3 University of Chinese Academy of Sciences, Beijing 100049, China

**Keywords:** carbon, attapulgite, nanocomposite, dye, adsorbent, reuse

## Abstract

Activated clay minerals have been widely used in the edible oil refining industry for decolorization of crude oil by adsorption, and so far many methods have been used to improve their decolorization efficiency. Herein, we successfully prepared a series of carbon/attapulgite (C/APT) composite adsorbents by a one-step in-situ carbonization process with natural starch (St) as the carbon source. It has been revealed that the adsorbent had better decolorization efficiency for crude palm oil than acid-activated APT. However, more than a million tons of decolorized waste is produced every year in the oil-refining industry, which was often treated as solid waste and has not yet been reutilized effectively. In order to explore a viable method to recycle and reuse the decolorant, the waste decolorant was further prepared into new C/APT adsorbents for the removal of dyes from wastewater, and then the dyes adsorbed on the adsorbent were used as the carbon sources to produce new C/APT adsorbents by a cyclic carbonization process. The results showed that the adsorbents prepared from the decolorized waste could remove more than 99.5% of the methylene blue (MB), methyl violet (MV), and malachite green (MG) dyes from the simulated wastewater with the dye concentration of 200 mg/L, and the C/APT–Re adsorbent consecutively regenerated five times using the adsorbed dyes as a carbon source still exhibit good adsorption efficiency for dyes. As a whole, this process opens a new avenue to develop efficient decolorants of palm oil and achieves recyclable utilization of decolored waste.

## 1. Introduction

Palm oil is a natural vegetable oil produced by pressing the fruits of oil palms. It is mainly composed of fatty acids, esterified with glycerol, and has the functions of reducing cholesterol, inhibiting thrombosis and preventing cardiovascular disease [1,2]. In recent years, palm oil has played an increasingly important role in cooking, food, fine chemicals, pharmaceuticals, and other fields, and has become the second largest edible vegetable oil in the world. However, crude palm oil is reddish in color due to high beta-carotene content, and there are hazardous impurities, such as peroxide, phospholipids, and others. Therefore, palm oil must be decolored before use to improve the color and quality of the oils [3,4].

At present, adsorption is the most effective way to remove color matters and other impurities from palm oil [5]. Acid-activated clay (i.e., activated montmorillonite (MMT)) as an effective decolorant has been widely used in palm oil refining industry [6,7]. However, there are still many problems that need to be solved. For instance, the commonly-used decolorization clay was produced by the activation of montmorillonite (MMT) with high concentrations of sulfuric acid solution (20–50 wt%), which need to be washed to neutral with large amounts of water. Thus, the product cost is high and the environmental pollution is serious. In addition, millions of tons of spent decolorization clays were generated every year in the edible oil refining industry, which was usually discarded, burned, used as land-fill as solid waste, or used as low-value filler [8,9]. Although the regeneration of spent decolorization clay by a calcination process has been reported [10,11,12], there are still no reports about the cyclic reuse of spent carbon/attapulgite decolorant.

MMT has been commonly used for the production of decoloring clay, which is a 2:1 type layered clay mineral with exchangeable interlayer cations [13]. It is composed of two SiO_4_ tetrahedron sheets, sandwiching an AlO_6_ octahedron sheet. Since MMT, itself, has no pore structure, in the preparation process of the decolorant the metal cations in the octahedral sheet must be etched by concentrated acid to create pores [14]. Different from MMT, attapulgite (APT) is a naturally-occurring rod-like nanoscale silicate clay mineral with rich pores and plentiful active silanol groups [15,16,17,18]. It has been widely used in many areas, such as colloid agents [19,20], adsorbents [21,22,23,24,25], carriers [26,27], reinforcing agents [28,29,30], animal feed [31,32,33], and hybrid functional materials [34,35]. Recently, APT as an adsorbent for decolorization of edible vegetable oil and removal of pollutants from waters has been of particular concern [36]. For example, APT has been widely used for decolorization of crude palm oil [37] or other edible oils [38], and a satisfactory decolorizing efficiency was achieved. Different from MMT, APT does not need to be activated with concentrated acid for preparation as an oil decolorant because it has intrinsic pores. In addition, it has been confirmed that the adsorption capability of APT can be improved by introducing carbon species onto it [39], which contribute to enhance the removal efficiency of color matter, gums, or other harmful matter from palm oil [40]. However, the carbon/APT (C/APT) composite would become decolorization waste after use, which contains about 10–21% normal grease.

In order to explore a sustainable way to develop efficient decolorant of palm oil, and cyclically reutilize the decolorization waste, we firstly prepared C/APT adsorbent with renewable, low-cost, and non-toxic starch (St) [41,42] as a carbon source and APT as the supporter, and used for the decolorization of crude palm oil. The resulting decolorization waste was regenerated by a one-step calcination process to produce new C/APT composites [43,44] and used for the removal of dyes (i.e., methylene blue (MB), methyl violet (MV), and malachite green (MG)) from polluted water. The dyes adsorbed on the composite were further used as carbon sources to produce C/APT-Re composites and cyclically used for dye adsorption. The main purpose of this paper is to develop a cyclic reusable C/APT composite and evaluate its reusability as a dye adsorbent, and finally pave a foundation for the sustainable utilization of waste clay adsorbents.

## 2. Results and Discussions

### 2.1. Structure and Characteristics of C/APT Composite

**Figure 1:**
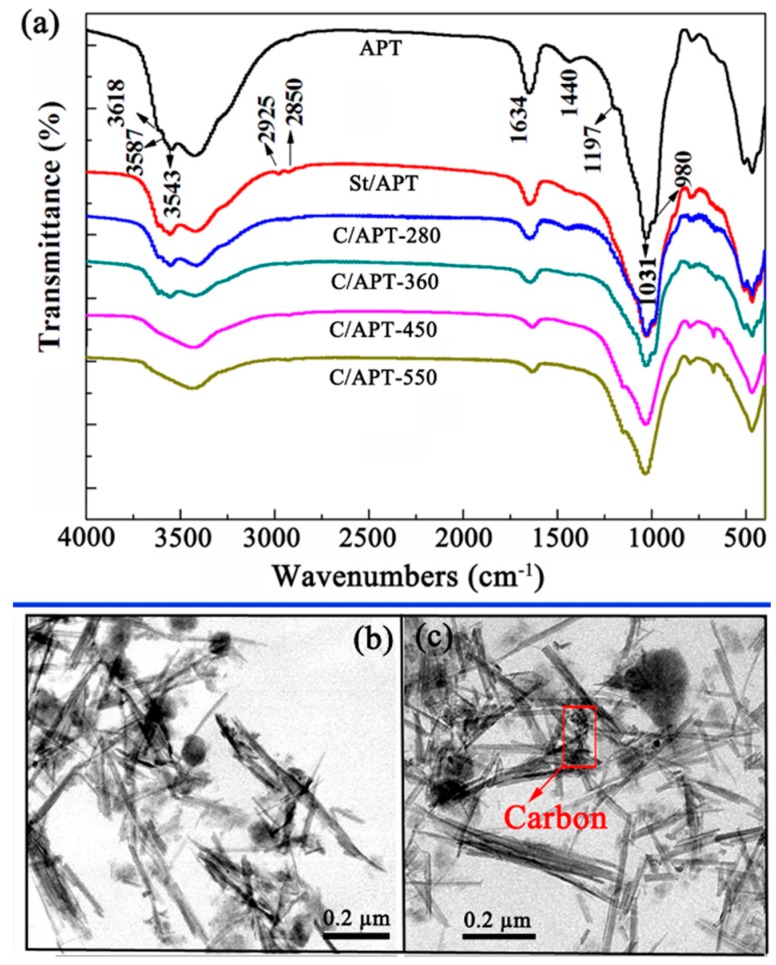
FTIR spectra of APT, St/APT precursor and the C/APT adsorbents prepared at different temperatures (**a**); and TEM images of APT (**b**) and C/APT-280 (**c**).

As shown in Figure 1a, two absorption bands at 2850 and 2925 cm^−1^ (C–H stretching vibration) appeared in the FTIR spectra of APT/St composites, indicating that St has been loaded onto APT [45]. With the increase of calcinations temperature, these absorption bands gradually disappeared (>280 °C), which revealed the combustion and decomposition of organic molecules. This phenomenon directly confirms that St has been carbonized after calcinations treatment.

As shown in Figure 1a, the absorption bands at 1197, 1088, 1028, and 980 cm^−1^ ascribed to the fingerprints of APT [46,47] disappeared gradually with the increase of the calcinations temperature, especially above 450 °C, due to the breakage of the tetrahedral crystal skeleton. The breakage of the tetrahedron would further destroy the octahedral structure of APT [47]. Likened to a domino effect, the breakage of the tetrahedral and octahedral structures would further cause the collapse of the pores and channels of APT.

**Figure 2:**
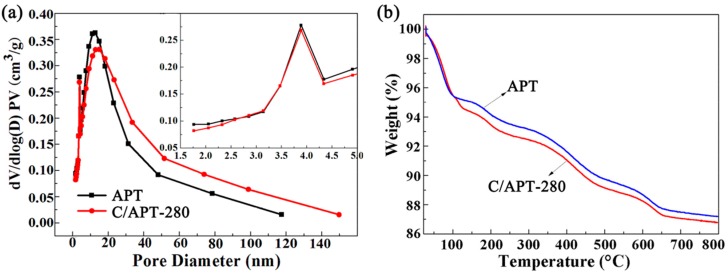
(**a**) Pore size distributions of APT and C/APT-280; and (**b**) TG curves of APT and C/APT-280 under an oxygen atmosphere.

As shown in Figure 1b, carbon nanoparticles were clearly observed on the surface of APT nanorods after the APT/St being calcined at 280 °C. The introduction of carbon might provide new pores serving as active adsorption sites to improve the decolorization efficiency of the as-prepared composite for crude palm oil. It was worth noting that the in situ-formed carbon increased the pore size distribution range (20–150 nm) of APT (Figure 2a), though the pore size distribution of APT and C/APT-280 were almost overlapping in the range of 3–5 nm. This also indicated that the modification of APT with St and the subsequent calcination process at 280 °C did not change the inner channels of APT.

Additionally, the formation of carbon species can also be verified by the TGA results. As shown in Figure 2b, the weight loss of C/APT-280 was larger than that of APT owing to the combustion and decomposition of carbon under an oxygen atmosphere. Consequently, the amount of carbon species loaded on APT can be calculated according to the weight loss ratio. The total weight losses of APT and C/APT-280 were 12.97% and 13.42%, respectively. Thus, the content of carbon species in the composite was about 0.45%. In other words, the amount of carbon species in C/APT was comparatively small. The color of the C/APT composites was deeper than that of APT (the inset in Figure 2b), and the C/APT composites appear grayish due to the presence of small amounts of carbon on APT.

### 2.2. Decoloring Efficiency

**Figure 3:**
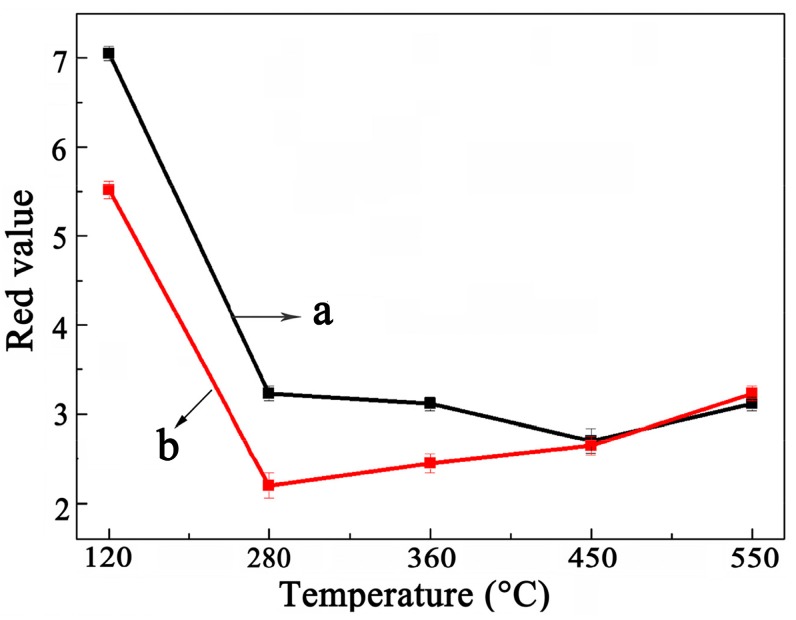
Change of red values of crude palm oil after being decolored by the APT (**a**) and C/APT (**b**) calcined at different temperatures (SD, *n* = 6).

As shown in Figure 3, the decolorization ability of APT and APT/St composite was initially enhanced with the increase in the calcinations temperature, and then it decreased. The C/APT composites prepared at the calcinations temperature of 280 °C show the best decolorization capability, but the higher calcinations temperature of 450 °C was needed to achieve the enhancement of the decolorization capability of natural APT. As for the C/APT decolorant, the optimal calcinations temperature is 280 °C, because too high a temperature would cause the decomposition of carbon species, and even lead to the damage of pores in APT [48].

As for natural APT, the thermal activation may selectively remove the water molecules from the channels of APT, which was favorable to improve the adsorption properties of APT [49,50]. However, the calcinations process at relatively higher temperature (>450 °C) may destroy the pores and channels of APT, which led to the decrease of the decolorization ability of APT to crude palm oil [48].

Usually, specific surface area of an adsorbent plays crucial roles in affecting the uptake of color matters from oil [51]. As shown in Table 1, the specific surface area of APT decreased after loading carbon, but the decolorization ratio for crude palm oil enhanced, indicating that the decolorization efficiency is not certainly dependent on the specific surface area. It has been found that the average pore size of C/APT-280 (8.33 nm) was larger than that of APT (7.33 nm). According to previous reports [52,53], an appropriate pore size was more favorable to remove the color matter from oil, and the suitable size for the absorption of carotene and chlorophyll was 3.5–15.5 nm and 6.0–7.5 nm, respectively. Therefore, the good decolorization efficiency of C/APT-280 is attributed to its appropriate pore size. After being decolored with acid-activated APT and C/APT-280, the red value of crude palm oil decreased to 3.2 and 2.2, respectively, indicating the C/APT-280 composite shows better decolorization capability than acid-activated APT. The carotenoid pigments, primary and secondary oxidation products ascribed to off-flavors [53,54], would be removed during the deodorization process.

### 2.3. Cyclic Regeneration of C/APT Composites

#### 2.3.1. Structure Characteristic of C/APT-Re Composite

As discussed above, the as-prepared C/APT adsorbent is highly efficient for the decolorization of crude palm oil, but it would become decolorization waste after use. If the decolorization waste is disposed of improperly, it not only did harm to our environment but also caused a waste of resources. Therefore, the sustainable utilization of decolorization waste was greatly significant. Since certain amounts of organic matter are present in the decolorization waste, it could be calcined at 300 °C to produce new C/APT adsorbent for adsorption of dyes from wastewater.

**Figure 4:**
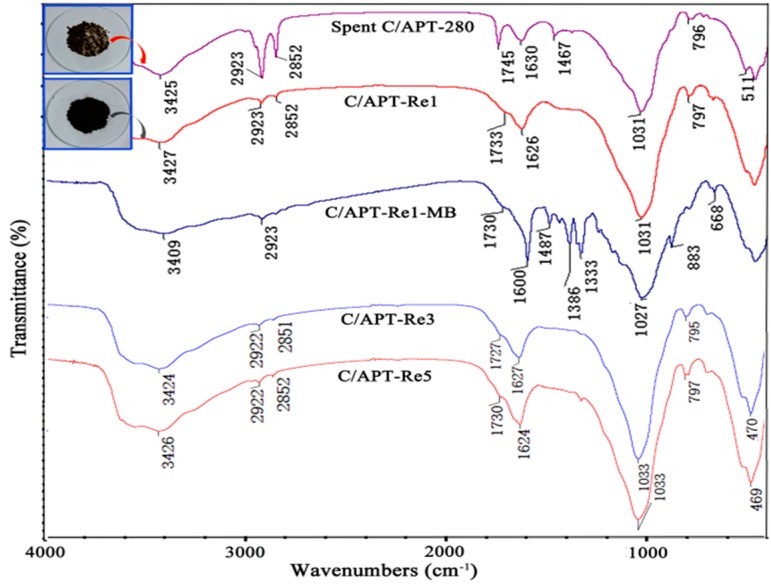
FTIR spectra of the spent C/APT-280 decolorant, C/APT-Re1 and C/APT-Re1-MB, C/APT-Re3 and C/APT-Re5. The inset shows digital photographs of spent C/APT-280 and C/APT-Re1, respectively.

As shown in Figure 4, after C/APT-280 decolorant was used for decoloring crude palm oil, the characteristic absorption bands at 2923 cm^−1^ (C–H stretching vibration of –CH_3_), 2854 cm^−1^ (C–H stretching vibration of –CH_2_–), 1467 cm^−1^ (methylene scissoring), and 1745 cm^−1^ (C=O stretching vibration) appeared [55], which proved the presence of organic species in C/APT-280. These absorption bands sharply weakened, and even disappeared, after calcination treatment, which confirm that the organic species have been carbonized and transformed as carbon species. Interestingly, the C=O stretching vibration band at 1733 cm^−1^, ascribed to the carboxyl groups, was still observed, which may be used as new active sites for the adsorption of dyes from polluted water [56]. The shift of this band from 1745 to 1730 cm^−1^ also confirmed the chemical changes of organic species during the calcination process.

**Figure 5:**
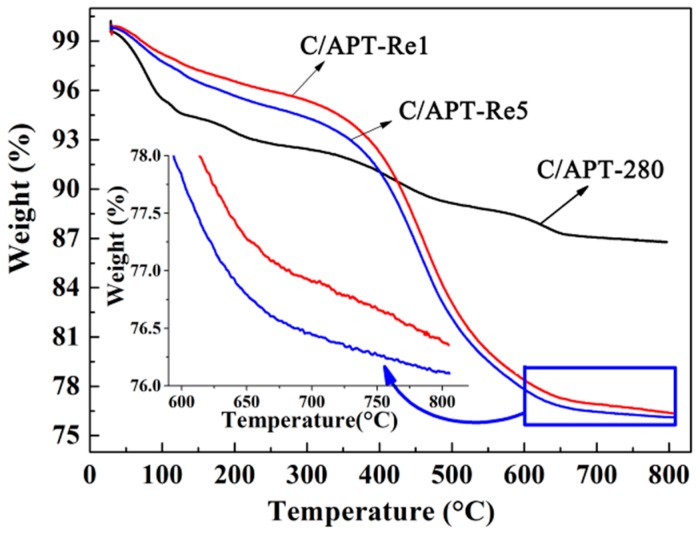
TG curves of C/APT-280, C/APT-Re1, and C/APT-Re5.

Moreover, it was observed from the digital photos (Figure 4) that the decolorization waste looks oily and its color is obviously deeper than C/APT-280, due to the adsorption of pigments or other substances from crude palm oil. After calcinations treatment, the oily substance was transformed as black powders, which visibly proved that the organic species were carbonized and the carbon species were regenerated on APT. Additionally, the amount of carbon species regenerated on APT could be calculated by TGA results. As shown in Figure 5, the total weight losses of C/APT-280 and C/APT-Re1 were 13.42% and 23.93%, respectively. Thus, the content of carbon species on C/APT-280 increased by 10.51% after used for decolorization of crude palm oil.

**Figure 6:**
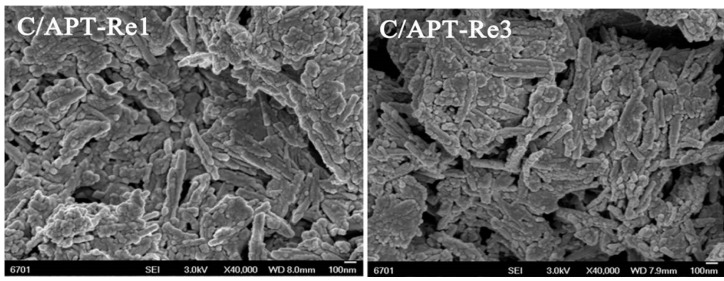

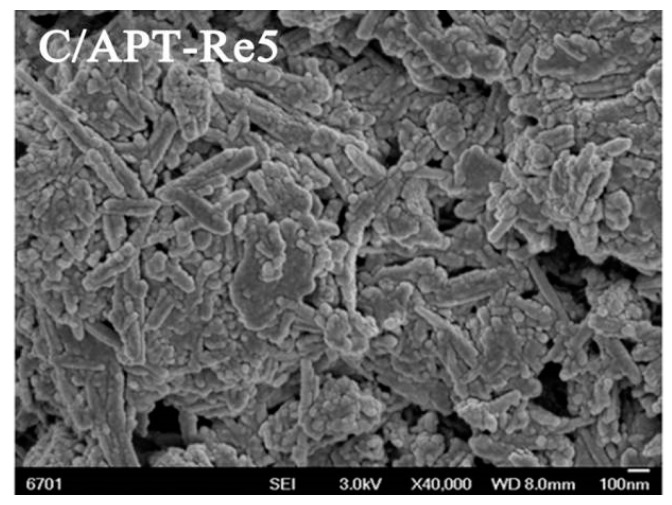
SEM images of C/APT-Re1, C/APT-Re3, and C/APT-Re5.

In this discussion, we can conclude that the spent C/APT-280 decolorant has transformed into new C/APT composites (marked as C/APT-Re1) after being calcined. The loading amounts of carbon species increased, while the active –COOH groups were simultaneously generated in the composite. As shown in Figure 6, the APT rod-like crystals were present in the form of bulk aggregates. With the increase in the regeneration times, the rod crystals became slightly thicker, and many particles appeared on the surface of rods due to the loading of carbon species.

#### 2.3.2. Adsorption Efficiency for Dyes

**Figure 7:**
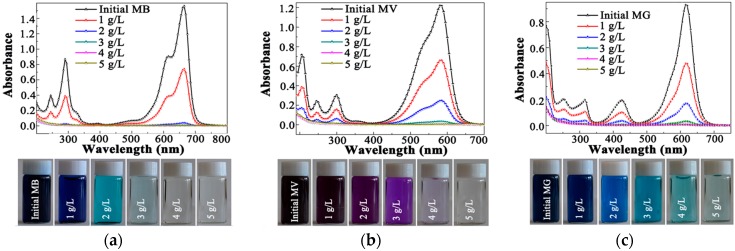
UV–VIS spectra of MB (**a**), MV (**b**), and MG solutions (**c**) after adsorption by C/APT-Re1; digital photographs of the MB, MV, and MG solutions.

The adsorption efficiency of C/APT-Re1 for cationic dyes MB, MV, and MG was evaluated. The adsorption experiments showed that C/APT-Re1 could remove more than 99.5% of the MB, MV, and MG molecules from 200 mg/L of dyes solution. The removal efficiency to each dye enhanced with increasing the dosage of C/APT-Re. The minimum dosage for the complete removal of MB, MV, and MG are 3.0, 4.0, and 5.0 g/L, respectively (Figure 7), indicating the as-prepared C/APT-Re1 adsorbent exhibited the best adsorption efficiency for MB. In addition, the effect of calcinations temperature, pH, ionic strength, and initial dye concentration on adsorption performance were investigated using MB as a model dye.

**Figure 8:**
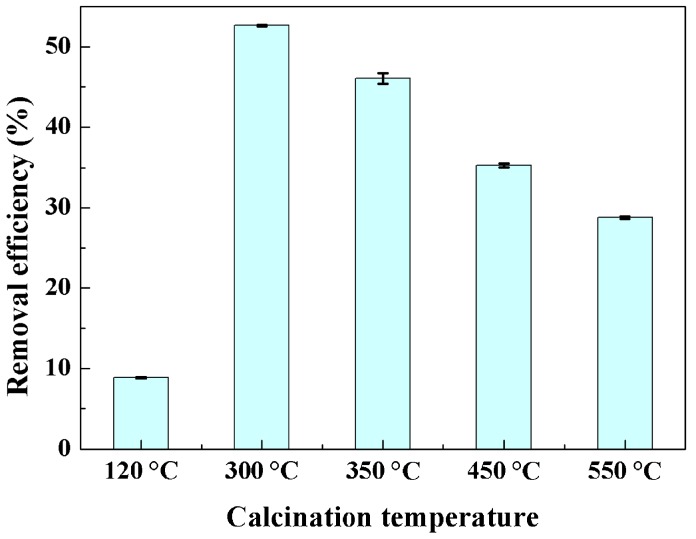
Effects of calcinations temperature on the adsorption efficiency of the used adsorbent (adsorbent dosage: 1.0 g/L).

The effect of calcination temperature on the adsorption of MB was studied and the result is shown in Figure 8. It was found that the removal ratio of the adsorbent for MB increased with increasing the calcinations temperature, reached the maximum value at 300 °C, and then decreased. When the calcinations temperature is lower than 150 °C, the organic matters cannot be carbonized [40], so the removal ratio is lower. With increasing the calcinations temperature, organic species adhered on the surface of APT was gradually carbonized and, thus, the pore structure parameters of samples increased, which contribute to improve the adsorption of regenerated adsorbent for MB. However, the high thermal treatment temperature would decompose the carbon species and lead to the collapse of the pores of attapulgite, which was not favorable to the adsorption of MB molecules. Therefore, the optimal calcinations temperature for the regeneration of spent adsorbent is 300 °C.

**Figure 9:**
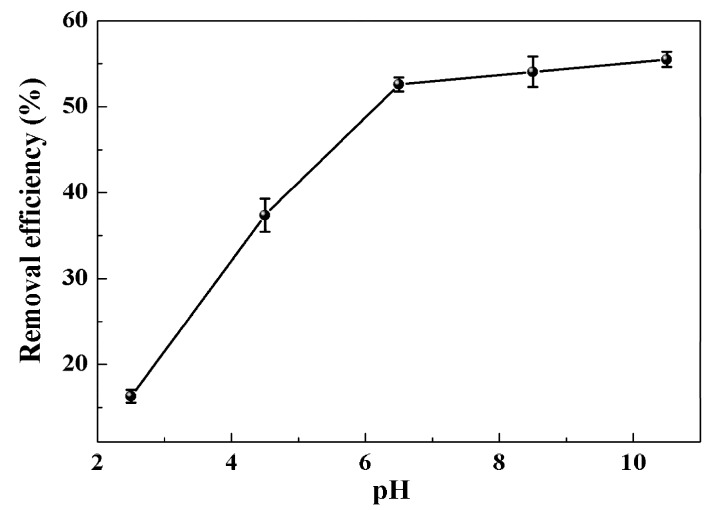
Effect of pH on the removal rate of MB (dosage of the adsorbents: 1.0 g/L; initial concentration: 200 mg/L).

According to the previous reports [57,58], the adsorption of an adsorbent for cationic dyes was mainly driven by electrostatic attraction and surface complexation. On the one hand, the adsorption of cationic dyes onto the C/APT nanocomposite is mainly attributed to the electrostatic attraction. The Zeta potential analysis showed that C/APT-Re1 is negatively charged (−19.23 mV), which is beneficial to capturing cationic dyes via electrostatic interaction [59]. Additionally, the evident influence of pH and ion strength on the adsorption capacity also implies that the electrostatic interaction plays important role in the adsorption process. As shown in Figure 9, the removal rate of MB increased with increasing the pH. According to the FTIR results (Figure 4), the active –COOH groups were formed in the composite after regeneration. In addition, the active Si–OH and Al–OH groups exist on the surface of APT are helpful to improve the adsorption [60]. At lower pH values, there is competition between the H^+^ ions and the cationic dye MB, and the negative charges on the adsorbent reduced with decreasing the pH values [61], which led to the reduction of the adsorption capacity for cationic dye. As pH increased, the dominating interaction would change to stronger electrostatic forces between the –X–O– groups (–X–OH, X represents of Si or Al or –CO) and the amine groups on the MB molecules and, thus, the removal rate of MB increased.

**Figure 10:**
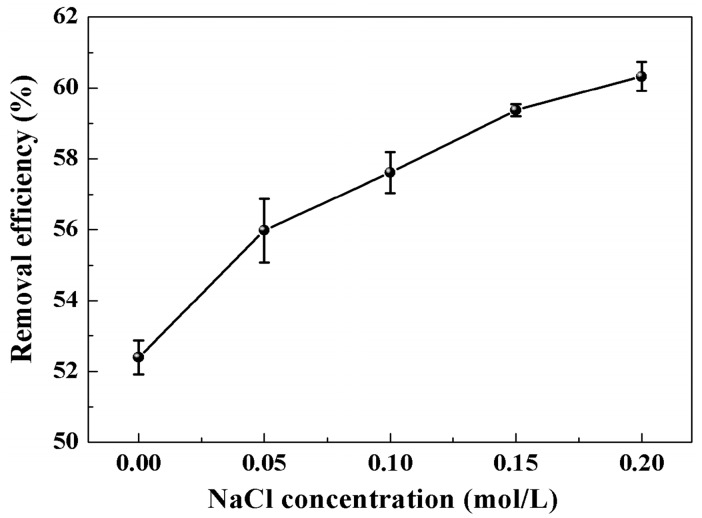
Influence of ionic strength on the removal amount of MB (dosage of the adsorbents: 1.0 g/L; initial concentration: 200 mg/L).

As shown in Figure 10, the removal rate of C/APT-Re1 for MB increased with the increase in the ionic strength. The results may be attributed to the following factors: (1) the addition of salt reduces the solubility of dye in the aqueous phase, thus improving the dye solubility in the adsorbent [62]; or (2) the addition of salt may cause the aggregation of MB cations due to a number of intermolecular forces, including van der Waals forces, ion dipole forces, and dipole–dipole forces, which occur between dye molecules in the solution [63]. The evident influence of pH and ion strength on the adsorption capacity implies that the electrostatic interaction plays an important role in the adsorption process.

Further, in order to determine whether other actions (i.e., surface complexation, hydrogen bonds), except electrostatic attraction, were involved in the adsorption process, the FTIR spectra of C/APT-Re1 before and after adsorption of MB were taken as examples and intensely discussed to obtain insight into the interaction between C/APT-Re1 and cationic dyes. As shown in Figure 4, the characteristic fingerprint of MB in the range of 1600–1200 cm^−1^ appeared in FTIR spectrum of C/APT-Re1-MB (the used C/APT-Re1, loaded with MB molecules). It was also found that the stretching vibration band of O–H groups at around 3427 cm^−1^ shifts to 3409 cm^−1^ after the adsorption of MB. In addition, the absorption bands of Si–O–H groups at 1031 cm^−1^ become blunt and broad, and slightly shifted from 1031 to 1027 cm^−1^ [46,60]. Simultaneously, the characteristic stretching vibration bands of C=O groups at 1733 cm^−1^ also shifted from 1733 to 1730 cm^−1^, and became blunt and broad. Additionally, the characteristic bands of MB at 1600 cm^−1^ (C=N stretching vibration), 1487 cm^−1^ (the first overtone N–H stretching vibration) and 1386 cm^−1^ (C–N stretching vibration) are obviously weakened and overlapped after MB was adsorbed onto C/APT-Re1. All these results suggested the strong interaction between the MB dye and C/APT-Re1, which is consistent with the research results of Bhattacharyya et al. [64].

**Figure 11:**
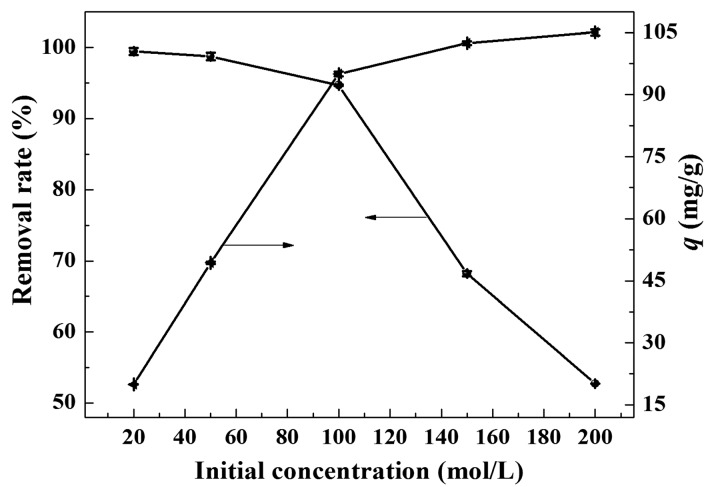
Effect of initial concentration on the MB adsorption (dosage of the adsorbents: 1.0 g/L).

The effect of initial dye concentrations on adsorption capacity was studied. As shown in Figure 11, the adsorption capacity of C/APT-Re1 for MB increased with increasing the initial concentration. The higher initial concentration will lead to a stronger driving force at the solid-liquid interface, which accelerates the diffusion of dye molecules onto the adsorbent [65]. Then, the increasing trend becomes flat until the adsorption saturation was reached when the maximum adsorption capacity was 105.1 mg/g. However, the removal rate of C/APT-Re1 for MB reduced with the increase of the initial concentration. This is because the adsorption sites are gradually saturated with the increase in the initial concentration.

**Figure 12:**
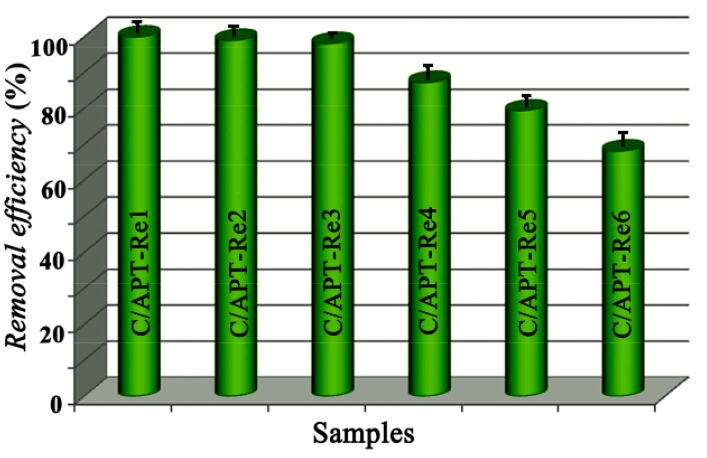
The removal efficiency of the re-generated C/APT-Re adsorbent for MB (dosage of the adsorbents: 2.0 g/L).

**Figure 13:**
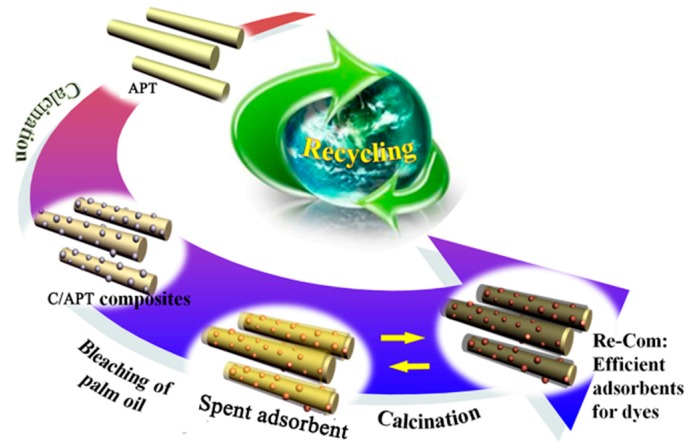
The cyclic regeneration and reusable route of C/APT decolorant, spent decolorant, and dye-loaded adsorbent.

Finally, to further reuse the spent dyes-loaded C/APT-Re1 composites, the MB-loaded C/APT-Re1 waste was taken as an example, and further recycled for another five times still a simple calcination method at 300 °C and used as adsorbents to remove MB from water. It was found from Figure 12 that the removal efficiency of C/APT-Re6 for MB in the 200 mg/L of initial solution is still higher than 75% after regeneration for five times, indicating that this composite has an excellent adsorption capacity. The slight decrease of adsorption capacity may be attributed to the excessive coverage of carbon species and the gradual agglomeration of APT nanorods, which decrease the active adsorption sites of the composites used for the removal of dyes. Accordingly, the results from the regeneration experiments show that the as-prepared C/APT composite can be used as an efficient recyclable adsorbent for the treatment of wastewater (Figure 13).

## 3. Materials and Methods

### 3.1. Materials

Natural APT, with the chemical composition of 58.38% SiO_2_, 8.55% Al_2_O_3_, 8.03% MgO, 6.63% Fe_2_O_3_, 3.47% CaO, 0.22% Na_2_O, and 1.24% K_2_O was from the Huangnishan Mine located on Xuyi County of Jiangsu Province (Jiangsu, China). Crude palm oil (originally produced from Malaysia) was provided by Dongma Oils and Fats (Guangzhou Free Trade Zone) Co. Ltd. (Guangzhou, China). Starch (St, USP grade) was purchased from Aladdin Reagent Corporation (Shanghai, China). MB (industrial grade), MV (N/A grade), and MG (AR grade) were all purchased from Sinopharm Chemical Reagent Co., Ltd. (Shanghai, China). The commercial decolorizer (CD) was provided by Taiko Marketing Sdn. Bhd, TMK (Pasir Gudang, Malaysia).

### 3.2. Preparation of C/APT Adsorbent from St and APT

**Scheme 1:**
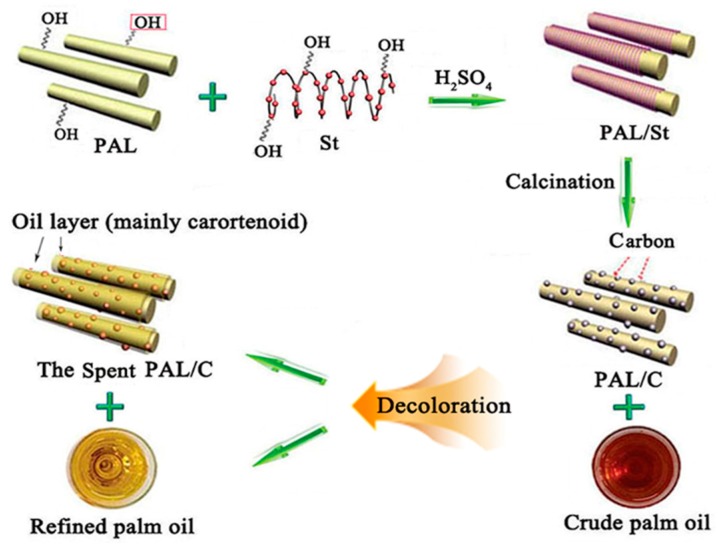
A schematic diagram of the preparation and decoloring procedure.

Natural APT powder (60 g) was fully dispersed in 4 wt% of aqueous solution of sulfuric acid at the solid-liquid ratio of 1:4 under continuous mechanical stirring. Then, 1 wt% of St was added into the dispersion and mechanically stirred at 800 rpm for 4 h. The resultant dispersion was passed through a 75 μm sieve to remove large grains of quartz. The solid product was separated from the dispersion by centrifugation at 4500 rpm and dried to a constant weight at 105 °C. The dry St/APT product was smashed and passed through a 200-mesh sieve to obtain the St/APT precursor. Finally, St/APT precursor was calcined at different temperatures (120, 280, 360, 450, and 550 °C) for 30 min to obtain the C/APT adsorbent. The natural APT was also calcined at the same conditions as a blank sample. According to the calcination temperatures, the as-prepared C/APT samples were marked as C/APT-120, C/APT-280, C/APT-360, C/APT-450, C/APT-550, respectively. A schematic diagram for the preparation and decoloring procedure was shown in Scheme 1.

### 3.3. Regeneration of the Spent C/APT Composites

The spent C/APT decolorant was recycled and calcined at 300 °C for 120 min to obtain a new C/APT composite, which was marked as C/APT-Re1. Again, the C/APT-Re1 adsorbents’ adsorbed dyes were also recycled and calcined at 300 °C for 120 min. According to the regenerated times, the recycled dye-loaded C/APT-Re1 adsorbents were marked as C/APT-Re2, C/APT-Re3, C/APT-Re4, C/APT-Re5, and C/APT-Re6, respectively.

### 3.4. Decoloring Test of Crude Palm Oil and Evaluation of Decoloring Capacity

To prevent the oxidation of oil, the decoloring test was carried out in a rotary evaporation meter with a rotation speed of 80 rpm and absolute pressure of 7 mbar. After being decolored, the mixture was cooled to 40–50 °C under vacuum, and filtered through a mid-speed filter paper (about 30–50 µm). The decolored oil was collected for Lovibond color, peroxide, and phospholipid analyses.

A Lovibond colorimeter (PFX-I series spectrocolorimeter, The Tinotometer Ltd., Amesbury, UK) was used to determine the Lovibond color by matching with a set of standard colored, numbered glasses, ranging in the scale from 0 to 70 red (R). A 2.5-cm vessel was used for measuring the refined oils, and the results were expressed as red values (R). Six parallel experiments were conducted and the averages were reported (±SD, *n* = 6).

### 3.5. Batch Adsorption Experiments

The adsorption experimental process is described as follows: 10, 20, 30, 40, 50 mg of adsorbent was added into 10 mL of MB, MV, and MG solution with an initial concentration of 200 mg/L (initial pH ≈ 6.5), respectively, followed by shaking at 180 rpm in a thermostatic shaker (THZ-98A, Shanghai Yi Heng Scientific Instruments Co., Ltd., Shanghai, China) at 30 °C for 2 h. The adsorbents were separated from the mixture by centrifugation with a speed of 5000 r/min for 10 min, and the absorbance of the solution before and after adsorption was determined using UV-visible spectrophotometer (UV 765, Precision and Scientific Instrument Co., Ltd., Shanghai, China).

For the evaluation of adsorption performance of C/APT-Re1~C/APT-Re6, 30 mg of adsorbent was mixed with 10 mL of MB solution (initial concentration of 200 mg/L, initial pH of 6.5), followed by shaking at 180 r/min in a thermostatic shaker (THZ-98A) at 30 °C for 2 h to reach the adsorption equilibrium. Afterward, the MB solution was separated from the mixture by centrifugation. The concentrations of MB before and after adsorption were determined using UV-VIS spectrophotometer (UV 765, Precision and Scientific Instrument Co., Ltd., Shanghai, China) at the maximum absorbance wavelength of 665 nm and calculated from the absorbance using a standard calibration curve. The removal efficiency and adsorption capacity of MB on the adsorbents are calculated by Equations (1) and (2), respectively:

*r* (%) = (*C*_0_ − *C*)/*C*_0_ × 100% (1)

*q = (C_0_* − *C_t_) × V/m* (2)

where *r* (%) is the adsorption ratio of the adsorbents; *V* (L) is the volume of MB solution; *m* (g) is the mass of adsorbent; *C*_0_ (mg/L) is the initial concentration of MB solution; and *C* (mg/L) is the concentration of MB in the solution at 2 h.

The main influence factors for the adsorption of MB onto the as-prepared composites, including pH values, ionic strength and initial concentration of MB solution were studied. The pH value of MB solution was adjusted with dilute NaOH or HCl solutions (0.1 mol/L) to a pH range 2–11 to study the effect of pH values on dye removal. Different amounts of sodium chloride were added into the MB solution during the adsorption process to study the effect of ionic strength on dye removal. A set of MB solutions with the initial concentration of 20–200 mg/L and pH~6.5 were adopted to test the effect of initial concentration.

### 3.6. Characterizations

The Fourier transform infrared (FTIR) spectra were recorded in the range of 4000–400 cm^−1^ on a NEXUS FTIR spectrometer (Nicolet, Madison, WI, USA) using the KBr pellets. Thermal gravimetric analysis (TGA) was tested from 35 to 900 °C on a Diamond TG-DTA 6300 thermal analyzer (PerKinElmer, Waltham, MA, USA) at the heating rate of 10 °C min^−1^ under an oxygen atmosphere. The specific surface area, pore volume and pore size distribution of the samples were measured on an Accelerated Surface Area and Porosimetry System (Micromeritics, ASAP2020, Atlanta, GA, USA) using N_2_ as an adsorbate at 77 K. The TEM images were taken using a JEM-2010 high resolution transmission electron microscope (HRTEM) (JEOL, Tokyo, Japan) at an acceleration voltage of 200 kV, and the sample was ultrasonically dispersed in anhydrous ethanol and dropped onto a grid before observation. The microscopic morphology was observed on a scanning electronic microscope (SEM, JSM-6701F, JEOL, Ltd., Tokyo, Japan) after the samples were fixed on copper sheets and coated with a gold film.

## 4. Conclusions

The C/APT composite adsorbent was prepared using St as the carbon source by the one-step calcination process, and the C/APT composite calcined at 280 °C showed the optimal decolorization efficiency for crude palm oil. The red value of crude palm oil decreased by 82.68% after being decolored with the C/APT adsorbent, which is better than that of a similar commercial decolorant (32.28%). The resultant decolorized waste was regenerated by the facile calcination method to derive new C/APT-Re adsorbent, which can remove more than 99.5% of the dyes from the dye solution with the initial concentration of 200 mg/L.

This work is a continuation of our systematic research works that focused on the comprehensive utilization of natural clay minerals, and showed the following advantages: (a) naturally abundant APT clay was used to develop a highly-efficient C/APT adsorbent with superior decolorization performance; (b) the decolorization waste was transformed to highly-efficient dye adsorbents; (c) the adsorbed dye can be used as new carbon sources to produce C/APT adsorbent; and (d) the sustainable regeneration utilization of the adsorbent was achieved in this way. In a word, the study not only developed a C/APT composite to improve the decolorization efficiency of APT for crude palm oil, but also achieved the sustainable use of the adsorbent and spent adsorbents.

## Figures and Tables

**Table 1:** Pore structure parameter of APT and C/APT-280.

Samples *	*S*_BET_/m^2^/g	SD	*S*_micro_/m^2^/g	SD	*S*_ext._/m^2^/g	SD	*V*_micro_/cm^3^/g	SD×10^4^	*V*_total_/cm^3^/g	SD× 10^3^	PZ/nm	SD
APT	192.4	1.5	49.9	0.5	142.5	2.0	0.03	3.1	0.35	8.4	7.33	0.1
C/APT-280	176.8	1.2	39.5	0.6	137.3	0.7	0.02	5.6	0.37	2.0	8.33	0.2

* The samples of the natural APT and APT/St composites are calcined at 280 °C for 30 min (SD, *n* = 3).
